# Active Sites on the CuCo Catalyst in Higher Alcohol Synthesis from Syngas: A Review

**DOI:** 10.3390/molecules29204855

**Published:** 2024-10-13

**Authors:** Chun Han, Jing Liu, Le Li, Zeyu Peng, Luyao Wu, Jiarong Hao, Wei Huang

**Affiliations:** 1 State Key Laboratory of Clean and Efficient Coal Utilization, Taiyuan University of Technology, Taiyuan 030024, China; 15552201638@163.com (C.H.); 18234133293@163.com (L.L.); david13834557950@163.com (Z.P.); ly21109@163.com (L.W.); 17735649473@163.com (J.H.); 2 Department of Chemistry and Chemical Engineering, Shanxi Polytechnic College, Taiyuan 030032, China; liujing951123@163.com

**Keywords:** higher alcohol synthesis, CuCo catalysts, stability, active sites, catalytic performance

## Abstract

Higher alcohol synthesis through the Fischer–Tropsch (F–T) process was considered a promising route for the efficient utilization of fossil resources could be achieved. The CuCo catalysts were proven to be efficient candidates and attracted much interest. Great efforts have been made to investigate the active sites and mechanisms of CuCo catalysts. However, the industrialized application of CuCo catalysts in this process was still hindered. The poor stability of this catalyst was one of the main reasons. This short review summarized the recent development of active sites on the CuCo catalysts for higher alcohol synthesis, including CuCo alloy particles, CuCo core–shell particles, and unsaturated particles. The complex active sites and their continual changes during the reaction led to the poor stability of the catalysts. The effect of active sites on catalytic performance was discussed. Furthermore, the key factors in fabricating stable CuCo catalysts were proposed. Finally, reasonable proposals were proposed for designing efficient and stable CuCo catalysts in higher alcohol synthesis.

## 1. Introduction

The gradual dwindling of fossil resources in the current world and the issues in the exploitation and utilization process, such as environmental contamination and resource waste, require the development of technologies for the production of valued chemicals from the efficient utilization of fossil resources [1]. In recent years, the concerns about the Fischer–Tropsch (F–T) process have increased as the chemical feedstock, such as hydrocarbons, olefins, and higher alcohols, could be directly converted from syngas (CO+H_2_) [2]. The production of syngas can be commercially obtained from coal, natural gas, biomass, and even organic waste and has reached commercialization [3,4,5,6]. Thus, the development of the F–T process is considered one of the promising routes for further development of the world.

Among the products in this process, higher alcohols (HA), which contain two or more carbons, have a wide range of applications, which can serve as fuel, fuel additives, and other platform molecules and attract much interest [7,8,9]. The Institut Francais du Petrole (IFP) first proposed that the CuCo catalyst showed excellent HA yield through the F–T process in the 1970s [10]. After that, a large amount of the work focused on facilitating the efficient CuCo catalysts has been made as the low-cost and rich reserves and the moderate operating conditions for high yields of HA [11]. Generally, the Cu and Co species are responsible for the CO non- and dissociative adsorption behaviors in this process, respectively. The combination of intermediates (CO* from Cu and CH_x_* from Co) is the key step to forming HA with the subsequent hydrogenation process [12]. It is considered that shortening the distance between these two species is the efficient way, which favor decreasing the barriers of CO insertion [13]. Consequently, constructing the bimetallic particles with intimate connections, such as alloy or core–shell particles, was the main strategy in recent years [14].

However, the industrialized application of this process is still hindered by the poor stability of the catalysts [15]. Actually, owing to the low solubility between Cu and Co [16], the preparation parameters have a significant effect on forming the species of components over the catalysts (as shown in Table 1). Moreover, unsaturated particles have been found over the catalysts in recent years, which are formed owing to the incomplete reduction [17,18,19,20], metal–support interaction [21], and carbonization in the reaction process [22], and have been identified as having the ability of CO absorption. Thus, it is implied that the active sites for HA synthesis over the CuCo catalysts are complex and have a tight correlation relationship with the stability of the catalysts.

Though the catalyst types [4,26], reaction mechanism [4,27,28], active sites [3,14], and preparation strategy [4,29,30] of the catalysts have been reviewed, the active sites over the CuCo catalysts have not been summarized comprehensively, and the reason for the poor stability of CuCo catalysts have rarely been discussed. Consequently, the objective of this short review was to address this gap by summarizing the active sites over CuCo catalysts from recent works. The overview started with the introduction of HA synthesis over CuCo catalysts and then moved to the classification of active sites for HA synthesis on CuCo catalysts. The emphasis has been placed on the discussion of the relationship between the active sites and stability property.

## 2. Summary of Active Sites for HA Synthesis over CuCo Catalysts

### 2.1. Alloy Particles

The CuCo alloy particles were confirmed as typical active sites for HA synthesis [31]. Prieto et al. [17] studied the structure–activity relationship of CuCo catalysts by combining DFT simulations and microkinetic modeling with experimental results. The simulation results showed that monometallic Cu_3_ and Co_3_ favor the formation of methanol and hydrocarbon, respectively, and the CuCo alloy phase acts as the active site for HA synthesis. Moreover, their experiment result also was consistent with the simulation results. Cao et al. [13] proposed a plausible reaction mechanism over CuCo alloy particles for HA synthesis, combining density functional theory (DFT) and micro-kinetic modeling. The CuCo alloy particles favored decreasing the required coverage of CH_x_* and CO, facilitating C-C coupling. Additionally, on the surface of CuCo (211), the low C-O dissociation barrier and the high rate of CH_x_-CO coupling were responsible for the high selectivity toward ethanol.

**Figure 1:**
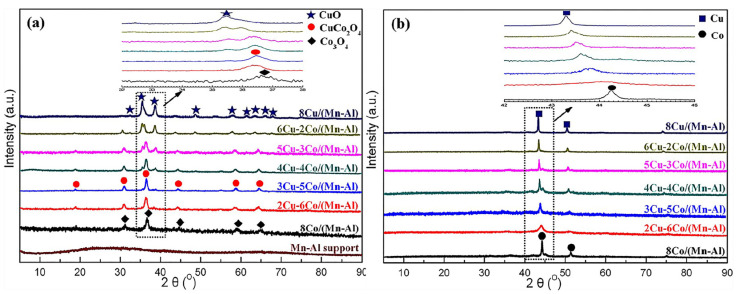
XRD patterns of (**a**) the calcinated and (**b**) reduced catalysts [38]. Copyright (2018) American Chemical Society.

Fabricating the precursor containing Cu and Co was the main route to form CuCo alloy particles as these two species were restrained into atom level [15,25,32,33,34,35,36,37]. The catalytic performance of CuCo alloy particles derived from various precursors is shown in Table 2. Zhao et al. [38] prepared a series of Mn-Al-supported CuCo catalysts with different Cu/Co ratios. Based on the characterization results (Figure 1), the dominant phase was the CuCo_2_O_4_ after calcination, and the uniformly CuCo alloy particles were obtained after reduction. Cao et al. [39] studied the catalytic performance of CuCo catalysts derived from layered double hydroxides (LDHs. The characterization results indicated that CuCo mixed oxides were reduced to form CuCo alloys, and the synergistic catalytic effect between Cu and Co in the alloy promoted the formation of HA. Li et al. [25] prepared a series of CuCo catalysts with different Co/Cu ratios encapsulated in KIT-6. After the stepwise pyrolysis, the results indicated that Co and Cu species were anchored into Cu_x_Co_3−x_O_4_ spinel oxide and the alloy phase was formed after reduction. The CuCo alloy particles were responsible for HA synthesis as a linear relationship between the surface proportion of alloy and yields of HA was displayed.

### 2.2. Core–Shell Particles

The core–shell structure was also responsible for HA synthesis because of the close connection between Cu and Co [43,44]. The catalytic performance of catalysts with core–shell structure is shown in Table 3. Subramanian et al. [43] conducted a DRIFTS study on the CO hydrogenation process over Cu@Co_3_O_4_. The results indicated that both dissociative and associative CO adsorption occurred on Cu@Co_3_O_4_. They concluded that the higher selectivity of HA was attributed to the existence of Cu@Co_3_O_4_.

Xiang et al. [23] investigated the effect of precursor activation on CuCo catalysts. It was found that the structure of CuCo was closely related to the reduction atmosphere. The Co@Cu particles were formed in H_2_, whereas a graphite-encapsulated onion-like structure was formed in CO. The catalyst activated by CO exhibited higher CO conversion (27.1%) than the catalyst activated by H_2_ (5.7%), which was attributed to the stronger CO dissociative adsorption ability of Co-rich species. Liu et al. [24] studied the catalytic performance of CuCo catalysts with Cu@Co and Co@Cu by adjusting reduction conditions. The reduction conditions were as follows: (1) heated in N_2_ to 600 °C, then reduced in H_2_ at 600 °C; and (2) heated from room temperature to 600 °C in H_2_. The characterization results showed that the catalyst reduced by method (1) displayed a Co@Cu structure (Cu shell and Co core), while the catalyst reduced by method (2) displayed a Cu@Co structure (Co shell and Cu core). The authors deemed that the Co@Cu structure was formed because of the aggregation of Cu species, as Cu has a lower surface energy than Co. For Cu@Co, it was ascribed to the lower reduction temperature of Cu and the effect of hydrogen spillover between Cu and Co. Meanwhile, the Co@Cu structure exhibited higher alcohol selectivity but lower CO conversion compared to Cu@Co.

### 2.3. Unsaturated Particles

In addition to the active sites mentioned above, many works found the presence of unsaturated particles on the CuCo catalysts. For example, Ye et al. [48] studied the catalytic performance of Na-doped catalysts. The X-ray photoelectron spectroscopy (XPS) results suggested that the Cu^+^, Cu^0^, Co^2+^, and Co^0^ species co-exist over the used catalysts. Meanwhile, the ratio of Cu^+^/Cu^0^ and Co^2+^/Co^0^ were affected by the additional amount of sodium. In recent years, large amounts of effort have been made to explore the role of unsaturated particles in HA synthesis, and their catalytic performance is shown in Table 4.

**Figure 2:**
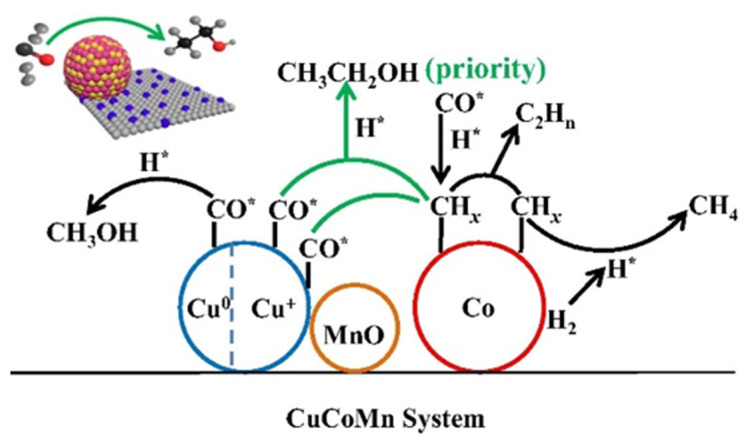
Schematic diagram of the mechanism of ethanol formation via the synergistic effect of Cu^+^-Co^0^ [49]. H* represented the dissociated H, CO* represented the non-dissociative adsorbed CO. Copyright (2019) Elsevier.

Sun et al. [49] applied Cu-Co-Mn catalysts in HA synthesis and observed significant alcohol selectivity (46.2%) and ethanol fraction (45.4%). The characterization results indicated that the Cu^+^ species existed on the catalyst owing to the strong interaction between Mn and Cu. They concluded that the outstanding performance was attributed to Cu^+^ species, which provided the absorbed CO was easier to participate in the CO insertion process than Cu^0^ [52,53]. They proposed that the synergistic effect between Cu^+^ and Co^0^ was responsible for ethanol formation (Figure 2).

The Cu^+^ and Cu^0^ also can participate in the process of HAS. Hofstadt et al. [54] suggested that the Cu^+^ and Cu^0^ favored the formation of C_2+_ alcohols. Gong et al. [55] studied the ethanol synthesis from syngas over Cu/SiO_2_ catalysts. As described, the Cu^0^0 was the sole active site for the activity of the catalysts, while the Cu+ was responsible for the conversion of intermediates. Wang et al. [53] studied the role of Cu^+^ and Cu^0^ species in CO hydrogenation reaction over Cu/SiO_2_ catalysts. They demonstrated that the Cu^+^ was responsible for the adsorption of methoxy and acyl species, while the Cu^0^ facilitated the H_2_ decomposition. Our groups made extensive efforts on the role of Cu species in CO hydrogenation reaction [56,57,58,59,60,61]. Zuo et al. [59] reported that the formation of ethanol needs the coexistence of Cu^+^ and Cu^0^ species, which would contribute to forming CH_3_ species. The DFT calculations suggested that the formed CH_3_ species were the key intermediate for ethanol formation as it would follow the CO insertion step.

**Figure 3:**
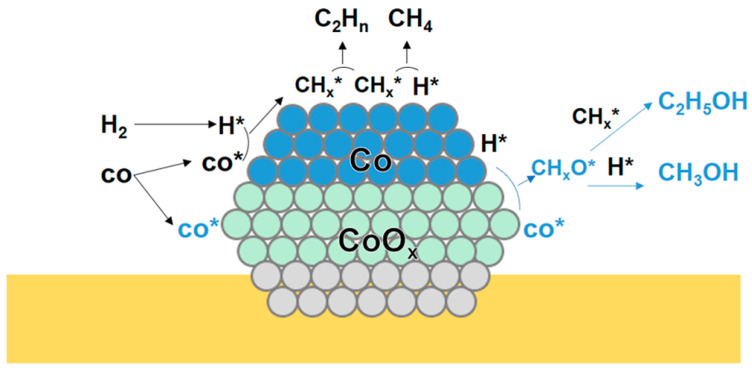
Schematic diagram of the mechanism of HA synthesis via the synergistic effect of CoO_x_-Co^0^ [62]. H* represented the dissociated H species. CO* represented the non-dissociative adsorbed CO species. CH_x_* represented the intermediates formed by the hydrogenation of dissociative adsorbed CO. Copyright (2018) American Chemical Society.

Sun et al. [62] investigated the CuCo catalysts derived from LDHs. A series of CuCoAl catalysts with different Cu/Co ratios were prepared. For the CoAl catalysts, the selectivity of total alcohol was 11.2%. The XPS and TEM results showed that CoO and Co(OH)_2_ coexist on the CuCo catalysts, which was ascribed to the metal–support interaction. For the CoAl catalysts, the selectivity of total alcohol was 11.2%. They speculated that the formation of HA may attributed to the Co^0^-Co^δ+^ pairs. Michel et al. [63] prepared the cobalt oxide-supported cobalt salt catalysts to convert the syngas into alcohols. After the reduction, the final state of the catalyst was Co-Al supported on CoAl_2_O_4_. The product formed in the Fischer–Tropsch synthesis was changed, and the production of alcohols was observed. They speculated that the CO insertion would occur on oxidized sites and the C-C coupling would occur on metallic sites. Chen et al. [64] prepared a series of Co/CeO_2_ with different Co content and applied in HA synthesis. During the reduction process, the characterization results showed that the Co_3_O_4_ was reduced into Co^2+^ and Co^0^ characteristics, evidenced that the Co^0^-CoO_x_ pairs were responsible for HA synthesis. The CO-DRIFTS indicated that Co^0^ favored CO dissociation, producing the CH_x_. The CO insertion occurred on Co^δ+^ species. They proposed that the synergistic effect between the Co^0^ and CoO_x_ species was responsible for HA formation (Figure 3).

Moreover, the unsaturated Co species could be formed by doping promoters, such as Ga [51,65,66,67,68,69,70], Mn [71], Ca [72] and Ce [73]. Gao et al. [66] prepared a series of gallium-doped CoGa/AC catalysts and applied them in HA synthesis. With the doping of Ga, the reduction temperature of Co_3_O_4_ increased, which favored adjusting the Co^2+^/Co^0^ ratio. The catalysts with the optimized ratio exhibited good alcohol selectivity (up to 30.3%). He et al. [68] reported that the formation of Co^2+^ was ascribed to the strong interaction between Ga and Co, to which the electrons would transform between Ga and Co. Meanwhile, the Co^2+^/Co^0^ pairs were responsible for HA formation.

Furthermore, the Co_2_C species also played an important role in HA synthesis. Nebel et al. [74] pretreated the CuCo catalysts into the CO atmosphere before the reaction. The characterization results illustrated that the Co_2_C was formed on the catalysts. They concluded that the improved HA selectivity and decreased CO_2_+methane selectivity contributed to the formation of Co_2_C. Extensive research has been made to investigate the role of Co_2_C [22,74,75,76,77,78,79,80,81,82,83,84]. Pei et al. [82] investigated the synergistic effect of Co-Co_2_C by combining DFT calculations with experimental results. In the experiment section, the Co_3_O_4_ sample without any support was prepared. After the carburization in the CO atmosphere, the Co_2_C was formed. The catalytic results illustrated the Co^0^-Co_2_C pairs were the active sites for HA synthesis. The DFT calculation results suggested that the Co_2_C was responsible for CO non-dissociative adsorption, whereas the Co^0^ was responsible for CO dissociative adsorption and the subsequent carbon-chain growth.

## 3. Discussion on Fabricating the Stable CuCo Catalysts

**Figure 4:**
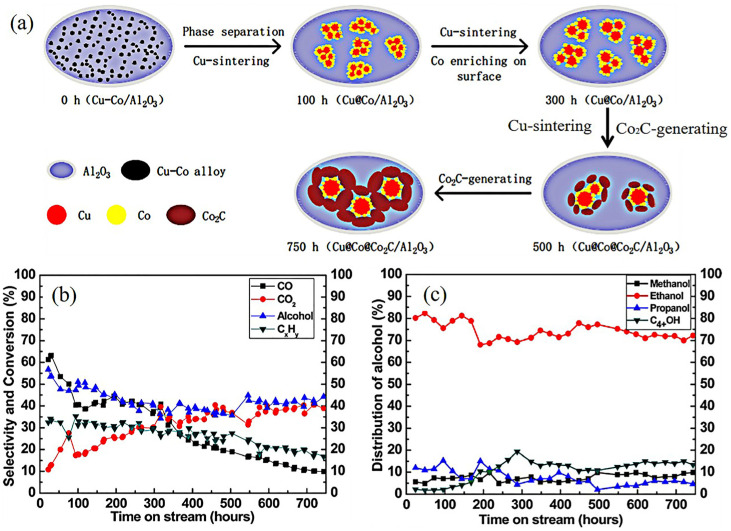
Structure evolution of CuCo alloy in HA synthesis. (**a**) The stability test of CuCo catalysts during 800 h [86]; (**b**) conversion of CO and selectivity of CO_2_, hydrocarbons, and total alcohols. (**c**) Distribution of alcohols. Copyright (2017) American Chemical Society.

As mentioned above, the active sites on the CuCo catalysts were complex. Research focused on stability studies found that the active sites were not stable during the reaction [85,86,87,88]. The Cu and Co showed poor miscibility in the Cu–Co alloys. Furthermore, the lower surface energy of Cu (1.934 J M^2^) than Co that of cobalt (2.709 J M^2^) could result in preferential localization of copper on the particle surface. Yang et al. [86] investigated the structure evolution of CuCo alloy particles in HA synthesis during 800 h (Figure 4). The characterization results show that the alloy particles decomposed into Cu and Co species at the beginning of the reaction (100 h). The core–shell particles gradually formed during 100–300 h. As the reaction continued, the sintering of Cu enlarged the size of the Cu@Co particles. Meanwhile, the Co_2_C was formed owing to the carbonization of Co. In 500–750 h, the Cu and Co NPs were wrapped by Co_2_C. The significant deactivation of catalytic performance was also observed in Figure 4b,c. Consequently, the transformation of the active sites during the reaction was the main reason for the poor stability of CuCo catalysts.

**Figure 5:**
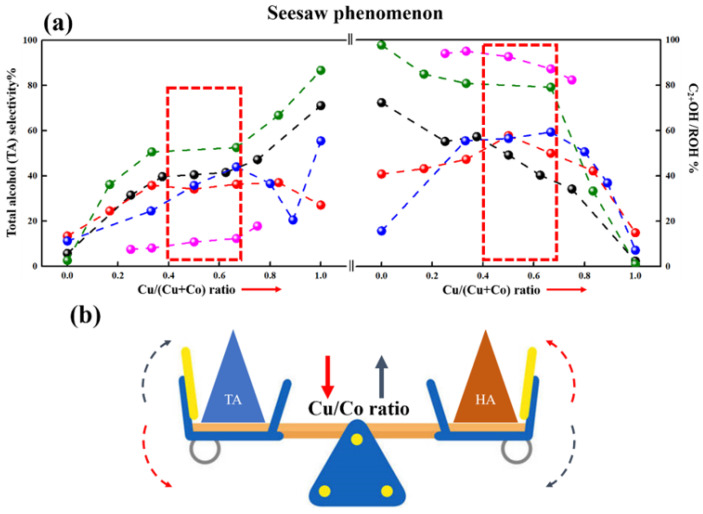
Catalytic performance of CuCo catalysts varying Cu/Co ratio. (**a**) Catalytic performance of CuCo catalysts varying Cu/Co ratio, (**b**) Schematic diagram of “Seesaw” phenomenon. (Note: the red and black arrow in (**b**) represents the decrease and increase in Cu/Co ratio, respectively).

The Cu/Co ratio played an important role in adjusting active sites over CuCo catalysts. Figure 5 summarizes the selectivity of total alcohol (TA) and HA with different Cu/Co ratios from recent studies. A “seesaw” phenomenon is clearly displayed in Figure 5a. For Cu-rich catalysts, TA selectivity was high, while HA selectivity was low. As the Cu/Co ratio decreased, the HA selectivity improved, but the TA selectivity decreased. Meanwhile, the proper TA and HA selectivity were performed over the catalysts with a ratio of nearly 1:1.

**Figure 6:**
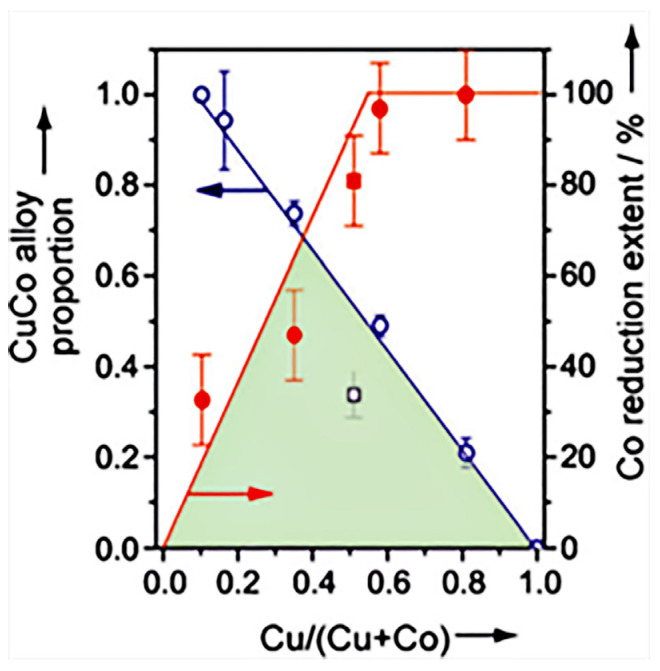
Effect of Cu/Co ratio on the active sites over Cu-Co catalysts [17]. Copyright (2014) John Wiley and Sons.

Prieto et al. [17] investigated the effect of the Cu/Co ratio on the active sites over the CuCoMo catalysts, and the evolution of the proportion of CuCo alloy was determined by XRD, and the extent of reduction of surface Co was determined by XPS (Figure 6). Obviously, a linear relationship was displayed between the proportion of the CuCo alloy phase and the Cu content, in which the CuCo alloy phase increased as the Cu content decreased. Conversely, the extent of reduction for Co species showed an increasing trend within the Cu/(Cu + Co) range of 0–0.5. The temperature-resolved X-ray diffraction and H_2_ consumption results indicated that Cu would be reduced at low temperatures over CuMo, while Co^2+^ was hard to be reduced over CoMo. However, with the existence of Cu, the Co^2+^ began to be reduced owing to the H_2_ dissociation on Cu. Thus, it was suggested that the dominant active sites on Cu-rich and Co-rich catalysts were different, and the effect of Cu/Co ratio needed to be taken into account to improve the stability.

For Cu-rich catalysts, owing to the hydrogen spillover effect, the existence of Cu facilitated the reduction of Co [89], which would weaken the role of unsaturated Co particles. However, owing to the different surface energy between Cu and Co, the segregation of Cu occurred. Liu et al. [90] investigated the size effect of Cu on HA synthesis over CuZnAl catalysts. The catalytic results showed that the HA selectivity was 8.9% over the catalysts with a smaller Cu size (11.8 nm), and the HA selectivity increased to 60.5% as the Cu size increased to 38.3 nm. They concluded that the larger Cu sizes favored forming HA. Zhang et al. [91] investigated the size dependence of C_2_ oxygenate formation from syngas on Cu cluster using the DFT method. The DFT results showed that the overall barrier differences between CHCO/CH_3_CO and CH_4_ formation were 58.9 and 11.6 kJ·mol^−1^ on the Cu_13_ cluster, and the value between CH_2_CO and CH_4_ was 21.9 kJ·mol^−1^ on Cu_38_ cluster. The values between CH_3_CO and CH_4_ were lower over the Cu_55_ cluster (−23.0 kJ·mol^−1^) than the Cu_13_ cluster. The different dominant products of Cu clusters with different sizes (C_2_ oxygenates for Cu_13_ and Cu_38_, CH_4_ for Cu_55_) suggested that Cu cluster size could affect the selectivity toward C_2_ oxygenates as the adsorption ability of intermediate was affected by adjusting the Cu cluster size. Thus, the crystallite size of Cu played an important role in HA synthesis. Consequently, preventing the segregation of Cu was one of the key factors to improve the stability of Cu-rich catalysts.

For Co-rich catalysts, it was proven that the Co-rich surface favored restraining the segregation of Cu. However, the separation of CuCo alloy particles, as mentioned above, resulted in the deactivation of catalytic performance (Figure 4). Moreover, the formation of Co_2_C would change the ratio of Co^0^/Co^δ+^, for which both Co^δ+^ and Co^0^ could be carbonized when exposed to syngas. Li et al. [74] investigated the effect of the Co^0^/Co_2_C ratio on the catalytic performance of HA synthesis. By changing the content of Na, a series of catalysts with different Co^0^/Co_2_C ratios were successfully prepared. They found that the catalytic activity and paraffin selectivity increased with the increase in Co^0^/Co_2_C, while the oxygenates selectivity displayed a volcano variation trend. They concluded that the proper Co^0^/Co_2_C ratio favored facilitating the CO insertion. Thus, the ratio of Co^0^/Co^δ+^ played an important role in HA synthesis. Consequently, preventing the separation of alloy particles and controlling the proper Co^0^/Co^δ+^ ratio were the main factors in improving the stability of Co-rich catalysts.

## 4. Conclusions and Perspectives

Higher alcohol synthesis through the Fischer–Tropsch (F–T) process was of significant importance for the efficient utilization of fossil resources. CuCo catalysts were proven to be efficient candidates and attracted much interest. Fabricating the stable CuCo catalysts remained a major challenge owing to the complex active sites on the catalysts. In this short review, the recent development of active sites on the CuCo catalysts was summarized, emphasizing the relationship between the active sites and stability. The evolution of active sites varied with the Cu/Co ratio. For Cu-rich catalysts, the role of Co^δ+^ was weakened owing to the hydrogen spillover effect. However, the segregation of Cu has always occurred. For Co-rich catalysts, the separation of CuCo alloy particles and the change in the Co^0^/Co^δ+^ ratio occurred during the reaction. The evolution of the active sites had a significant effect on the catalytic performance of higher alcohol synthesis. Thus, to fabricate the stable CuCo catalysts, the Cu/Co ratio needed to be taken into account. With the development of the catalysts designing concept and reaction mechanism, the following issues are expected to be addressed.

Firstly, the impact of active sites on CO adsorption ability and their continual changes during reactions make it challenging to detect evolution in real time. Since alcohol distribution is closely related to active site behavior, studying the relationship between catalytic performance, alcohol distribution, and active sites is crucial for developing effective methods to explore catalytic mechanisms.

Secondly, the catalytic mechanism remains unclear due to the diverse forms of unsaturated Co species, such as CoO, Co_2_C, and Co^δ+^. Advanced characterization techniques should be used to identify the CO adsorption and insertion abilities of these species.

Finally, the “seesaw” phenomenon should be investigated. The Cu-rich catalysts exhibit higher total selectivity and lower HA selectivity. The effect of the Cu^0^/Cu^+^ ratio and the crystallite size of Cu should be further revealed. The Co-rich catalysts exhibit lower total selectivity and higher HA selectivity. Studies on preventing the separation of alloy particles and controlling the proper Co^0^/Co^δ+^ ratio should be further investigated.

We hope that this review will inspire the designing of efficient and stable CuCo catalysts and foster further advancements in HA synthesis. Moreover, we eagerly anticipate the emergence of innovative research dedicated to overcoming the challenges of HA synthesis.

## Figures and Tables

**Table 1:** Effect of preparation parameters on the species of components over CuCo catalysts.

Preparation Parameter	Operating Condition	Surface Component	Refs.
Reduction atmosphere	H_2_ or Ar	Cu shell and Co core	[23]
	CO or Syngas	Graphitic carbon shell and CuCo core	
Reduction method	Heated in N_2_ to 600 °C and reduced in H_2_ at 600 °C	Cu shell and Co core	[24]
	Heated from room temperature to 600 °C in H_2_	Co shell and Cu core	
Cu/Co ratio	Cu_1_Co_1_	CuO (70.2 molar%) Cu_x_Co_3−x_O_4_ (29.8 molar%)	[25]
	Cu_1_Co_2_	CuO (54.5 molar%) Cu_x_Co_3−x_O_4_ (45.5 molar%)	
	Cu_1_Co_3_	CuO (38.5 molar%) Cu_x_Co_3−x_O_4_ (61.5 molar%)	
	Cu_1_Co_4_	CuO (45.8 molar%) Cu_x_Co_3−x_O_4_ (54.2 molar%)	

**Table 2:** Catalytic performance CuCo catalyst with alloy phase derived from precursors.

Precursor	Catalyst	T (°C)	P (MPa)	H_2_/CO ^a^	GHSV (mL g^−1^ h^−1^) ^b^	X_CO_ (%) ^c^	$S_{{\mathrm{CO}}_{2}}$ (%) ^d^	S_ROH_ (%) ^e^	C_2_+OH (%)	Ref.
Perovskite	Co–Cu/ZrO_2_-La_2_O_3_	310	3	2	3900	35.3	7.1	43.4	82.3 ^f^	[24]
	Cu-Co/GE-LFO	300	3	2	3900	49.7	7.9	56.9	47.2 ^g^	[40]
	CuCo/Al_2_O_3_/CFs	220	3	2	3900	38.5	1.4	47.3	96.0 ^f^	[41]
	CuCoAl	270	2.5	2	7500	36.2	0.5	40.7	54.9 ^h^	[42]
MOFs	Co_4_Cu_1_-Z	270	3	1	4800	39.7	2.0	35.6	73.0 ^f^	[33]

^a^ Molar ratio; ^b^ the gas hourly space velocity; ^c^ CO conversion; ^d^ CO_2_ selectivity; ^e^ total alcohol selectivity; ^f^ weight percentage of HA in alcohols (wt%); ^g^ HA selectivity; and ^h^ percentage of HA in alcohols (Cmol%).

**Table 3:** Catalytic performance of CuCo catalyst with core–shell structure.

Catalyst	T (**°C**)	P (MPa)	H_2_/CO ^a^	GHSV (mL g^−1^ h^−1^) ^b^	X_CO_ (%) ^c^	$S_{{\mathrm{CO}}_{2}}$ (%) ^d^	S_ROH_ (%) ^e^	C_2_+OH (%)	Ref.
Cu/Co	300	6	2	10,000	35.6	44.4	14.1	56.2 ^f^	[44]
Cu@(CuCo-alloy)/Al_2_O_3_	220	2	2	2000	21.5	7.2	50.6	80.8 ^f^	[45]
Co@Cu-2	230	2	2	/	/	30.3	33.0	29.6 ^g^	[46]
CoCu0.2	260	6	2	3000	32.1	1.4	14.1	51.2 ^f^	[47]

^a^ Molar ratio; ^b^ the gas hourly space velocity; ^c^ CO conversion; ^d^ CO_2_ selectivity; ^e^ total alcohol selectivity; ^f^ weight percentage of HA in alcohols (wt%); and ^g^ HA selectivity.

**Table 4:** Catalytic performance of catalysts with unsaturated particles.

Active sites	Catalyst	T (^°^C)	P (MPa)	H_2_/CO ^a^	GHSV (mL g^−1^ h^−1^) ^b^	X_CO_ (%) ^c^	$S_{{\mathrm{CO}}_{2}}$ (%) ^d^	S_ROH_ (%) ^e^	C_2_+OH (%)	Ref.
Cu^+^-Co^0^	CuCoMn	270	2.5	2	7500	29.7	/	46.2	70.4 ^f^	[49]
Cu^+^-Cu^0^	CuZnAl	250	4.5	2	/	12.4	49.0	30.7	48.0 ^f^	[50]
Co^0^-Co^δ+^	Co_3_O_4_-Ga_2_O_3_	310	3	2	3900	10.5	4.7	45.6	87.6 ^f^	[51]
	LaCo_0.9_Mn_0.1_O_3_	250	3	2	4000	21.5	3.1	25.8	75.7 ^f^	[22]

^a^ Molar ratio; ^b^ the gas hourly space velocity; ^c^ CO conversion; ^d^ CO_2_ selectivity; ^e^ total alcohol selectivity; and ^f^ weight percentage of HA in alcohols (wt%).

## Data Availability

The data presented in this study are available upon request from the corresponding author.
